# What parameters from ^18^F-FDG PET/CT are useful in evaluation of adrenal lesions?

**DOI:** 10.1007/s00259-014-2844-1

**Published:** 2014-07-16

**Authors:** Jolanta Kunikowska, Renata Matyskiel, Sadegh Toutounchi, Laretta Grabowska-Derlatka, Łukasz Koperski, Leszek Królicki

**Affiliations:** 1 Nuclear Medicine Department, Medical University of Warsaw, ul. Banacha 1 a, 02-097 Warsaw, Poland; 2 Department of Radiology, Medical University of Warsaw, Warsaw, Poland; 3 Department of General Surgery and Chest Diseases, Medical University of Warsaw, Warsaw, Poland; 4 Department of Pathology, Medical University of Warsaw, Warsaw, Poland

**Keywords:** ^18^F-FDG, PET/CT, Adrenal lesion

## Abstract

**Purpose:**

Prior studies have suggested that ^18^F-FDG PET/CT can help characterize adrenal lesions and differentiate adrenal metastases from benign lesions. The aim of this study was to assess the value of ^18^F-FDG PET/CT for the differentiation of malignant from benign adrenal lesions.

**Methods:**

This retrospective study included 85 patients (47 men and 38 women, age 63.8 ± 10.8 years) who had undergone ^18^F-FDG PET/CT (60 min after injection 300 – 370 MBq ^18^F-FDG; Biograph 64 scanner) for evaluation of 102 nonsecreting adrenal masses. For semiquantitative analysis, the maximum standardized uptake value (SUVmax), adrenal to liver (T/L) SUVmax ratio, mean CT attenuation value and tumour diameter were measured in all lesions and compared with the pathological findings.

**Results:**

Malignant adrenal tumours (68 % of evaluated tumours) had a significantly higher mean SUVmax (13.0 ± 7.1 vs. 3.7 ± 3.0), a higher T/L SUVmax ratio (4.2 ± 2.6 vs. 1.0 ± 0.9), a higher CT attenuation value (31.9 ± 16. 7 HU vs. 0.2 ± 25.8 HU) and a greater diameter (43.6 ± 23.7 mm vs. 25.6 ± 13.3 mm) than benign lesions. The false-positive findings were tuberculosis and benign phaeochromocytoma. Based on ROC analysis, a T/L SUVmax ratio >1.53, an adrenal SUVmax >5.2, an attenuation value >24 HU and a tumour diameter >30 mm were chosen as the optimal cut-off values for differentiating malignant from benign tumours. The areas under the ROC curves for the selected cut-off values were 0.96, 0.96, 0.88 and 0.77, respectively. A multivariate logistic regression model revealed that the T/L SUVmax ratio was an independent prognostic factor for malignancy (*p* < 0.001); a CT attenuation value of >25 HU and a tumour diameter >30 mm had no additional individual importance in the diagnosis of malignancy.

**Conclusion:**

Using a T/L SUVmax ratio >1.53 and an adrenal SUVmax >5.2 in ^18^F-FDG PET/CT led to high diagnostic sensitivity, specificity and negative predictive value for characterizing adrenal tumours. The diagnostic accuracies of the two parameters were comparable, but T/L SUVmax ratio was an independent predictor of malignancy.

## Introduction

Approximately 50 % of adrenal lesions in patients with a primary known extraadrenal malignancy are metastatic. However, benign adrenal tumours may occur in the general population and in patients with cancer. In oncological patients, distinguishing benign from metastatic adrenal tumours is often critical in determining the stage of disease, in predicting prognosis and in determining optimal management [1–3]. Conventional imaging methods used to characterize adrenal lesions, such as unenhanced CT attenuation, enhancement washout CT or chemical-shift MRI, have some limitations in predicting malignancy, indicating the need for new imaging technologies. ^18^F-FDG PET/CT offers the opportunity to assess glucose cell metabolism that is reprogrammed toward increased glycolysis in many cancers [4, 5]. Metabolic imaging with PET is not only complementary to imaging with conventional modalities but also may be more sensitive because alterations in the metabolism of cancer cells may precede gross anatomical changes [3, 6, 7]. On the other hand, ^18^F-FDG accumulates not only in malignant adrenal tumours but also in 5 – 10 % of benign tumours that mimic metastasis and limit the specificity of ^18^F-FDG PET/CT imaging. In published studies, false-positive ^18^F-FDG PET/CT findings mainly represent adrenal adenomas, benign phaeochromocytomas, endothelial cysts, and inflammatory and infectious lesions [8, 9].

Different algorithms for distinguishing benign from malignant tumours with ^18^F-FDG PET/CT have been proposed. The visual criteria seem to be subjective and inconsistent, especially for borderline adrenal activity [3, 7, 10–14]. Recent studies have shown that the adrenal to liver (T/L) maximum standardized uptake value (SUVmax) ratio is a more accurate and reliable parameter than SUVmean and SUVmax, but data on the routine use of the T/L SUVmax ratio are still insufficient [5, 15, 16]. The aim of this study was to assess the value of ^18^F-FDG PET/CT for differentiation of malignant from benign adrenal lesions. We explored the possibility of further improving the performance of ^18^F-FDG PET/CT using the T/L ratio for characterization of adrenal tumours. The results were expressed in relation to histology as the reference method.

## Materials and methods

We performed a retrospective analysis of preoperative ^18^F-FDG PET/CT scans obtained in 85 patients (47 men and 38 women; mean age 63.8 ± 10.8 years, range 33 – 86 years) with nonsecreting unilateral or bilateral adrenal masses identified on CT.

The examinations were performed from January 2009 to December 2012 in the Nuclear Department of the Medical University of Warsaw. Written informed consent was obtained from all patients. Within 3 months of PET/CT imaging, all patients underwent adrenalectomy (68 unilateral and 17 bilateral). Of the 85 patients, 41 had histopathologically proven malignancies, as follows: lung cancer (10), colorectal cancer (20), larynx carcinoma (2), breast cancer (6), cervix cancer (2) and melanoma (1). The remaining 44 patients had a suspected cancer with adrenal metastases.

### PET/CT protocol

The patients fasted for at least 6 h before the examination and blood glucose measurements were obtained in all patients before the administration of ^18^F-FDG. Blood glucose levels in all patients were less than 150 mg/dL (8.33 mmol/L). The PET/CT examinations were performed 60 min after intravenous injection of 300 to 370 MBq ^18^F-FDG from the skull base to the upper thighs on a Biograph 64 TruePoint PET/CT scanner (Siemens Medical Solutions, Knoxville, TN) using three-dimensional mode. All patients were requested to drink 1.5 L of water for hydration and empty their bladder before the PET/CT examination. Patients were positioned supine with their arms raised according to standard CT practice. Initially, low-dose CT scans were performed using a 64-detector helical CT scanner primarily for attenuation correction. A continuous low-dose CT scan was acquired in spiral mode using 120 kV, 170 mAs, slice thickness 2 mm, and pitch 0.8. No intravenous or oral contrast agent was administered. The PET study was performed immediately after the CT scan with the patient in the same position covering an area identical to that covered by the CT scan with 2 min per bed position (six or seven bed positions depending on the size of the patient). PET image data were reconstructed on a 168 × 168 matrix using the ordered-subsets expectation maximization algorithm (two iterations, 14 subsets) and corrected for attenuation using the CT data. The PET/CT images (half-body-attenuated and non-attenuated PET, CT and fused images) were transferred to a multimodal work station (Syngo (TrueD); Siemens Medical Solutions) for analysis.

### ^18^F-FDG PET/CT and CT imaging data

Tumours were quantitatively assessed by a nuclear physician with 5 years experience, and involved measuring SUVmax of adrenal lesions and the T/L ratio. The SUVmax of ^18^F-FDG in adrenal tumours was measured by drawing a region of interest (ROI) that encompassed the central two-thirds of the adrenal mass but did not include any peripheral areas to avoid partial volume effects. The liver SUVmax values were determined from a ROI drawn on a large homogeneous region of ^18^F-FDG distribution within the right hepatic lobe at the same axial level as the adrenal lesions, avoiding areas of large vessels and parenchymal abnormalities. The T/L SUVmax ratio was calculated with these results by dividing the adrenal tumour SUVmax by the liver SUVmax value.

An experienced radiologist analysed the CT data obtained from PET/CT imaging. Native CT of the abdomen with a slice thickness 5 mm was performed. Density is expressed in Hounsfield units. The maximum diameter and mean unenhanced attenuation (Hounsfield units) was calculated for each adrenal lesion.

### Morphological examinations

Surgically resected specimens were fixed in 10 % neutral buffered formalin and routinely processed for paraffin embedding. The sections were cut to a thickness of 4 – 5 μm, stained with haematoxylin and eosin and examined under a light microscope. Additionally in some cases immunohistochemical and histochemical studies were performed. Pathologists with specific training and expertise in adrenal pathology made all the morphological diagnoses.

### Statistical methods

Parameters were characterized in terms of the mean (±SD). To determine if there was a statistically significant difference between malignant and benign tumours in terms of adrenal SUVmax, T/L SUVmax ratio, attenuation value and tumour diameter, the Mann-Whitney *U* test was used. A *p* value of <0.05 was considered statistically significant.

We used univariate logistic regression models to assess predictors of malignancy. The predictive power of the univariate logistic regression models with continuous predictors was assessed by receiver operating characteristic (ROC) analysis. ROC curves indicating the ability to discriminate malignant from benign tumours were plotted for adrenal SUVmax, T/L SUVmax ratio, attenuation value and diameter, and optimal cut-off values were chosen. ROC curves were compared based on their area under the curve (AUC) as a summary measure of diagnostic accuracy.

A multivariate logistic regression model was used with adrenal SUVmax, T/L SUVmax ratio, CT attenuation value and tumour diameter categorized according to the established cut-off values as predictors to examine the independence of their relationship with the presence of malignant lesions. Odds ratios (OR) and 95 % confidence intervals (CI) for significant predictors are presented. A 5 % level of significance was used. Calculations were performed using Stata v10 (Stata Statistical Software, release 10; Stata Corporation, College Station, TX).

## Results

**Fig. 1:**
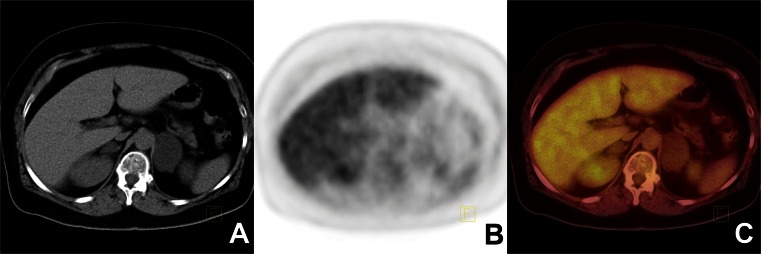
^18^F-FDG PET/CT findings in a 55-year-old women with disseminated metastasis from an unknown primary cancer (**a** CT image, **b** PET image, **c** fused PET/CT image). The ^18^F-FDG PET/CT image shows low ^18^F-FDG uptake. The SUVmax of the enlarged right adrenal tumour was 2.3 and the attenuation value −11 HU; the liver SUVmax was 4. These findings were consistent with a lipid-rich adenoma

**Fig. 2:**
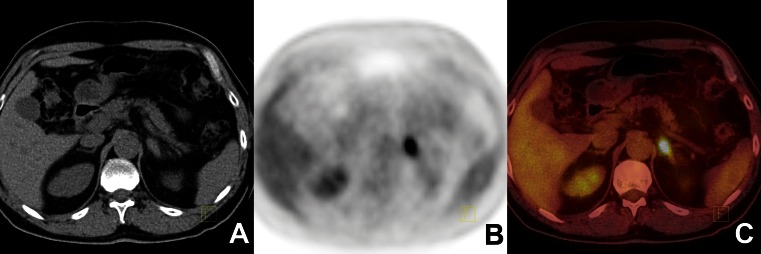
Adrenal metastasis from lung cancer. ^18^F-FDG PET/CT findings in a 53-year-old man with lung cancer (**a** CTimage, **b** PET image, **c** fused PET/CT image). The ^18^F-FDG PET/CT image shows increased uptake of ^18^F-FDG in a left adrenal tumour. The SUVmax of the tumour was 10.1 and the attenuation value 21 HU; the liver SUVmax was 3.1. The histopathological diagnosis was metastasis from lung cancer

**Fig. 3:**
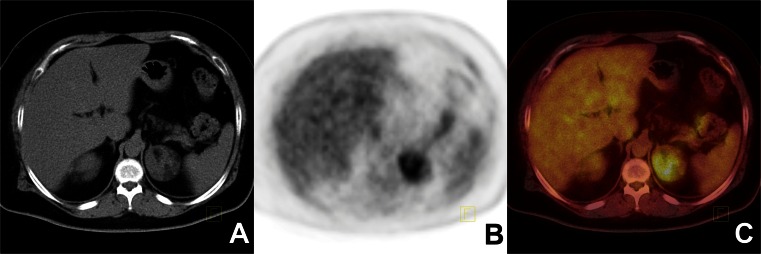
Malignant transformation of adrenal angiomyolipoma. ^18^F-FDG PET/CT findings in a 59-year-old woman with an unknown primary cancer (**a** CT image, **b** PET image, **c** fused PET/CT image). The ^18^F-FDG PET/CT image shows increased ^18^F-FDG uptake in a left adrenal tumour. The SUVmax of the tumour was 6.3 with attenuation values in the range −57 to 77 HU (mean 16 HU); the liver SUVmax was 3.6. This tumour was classified correctly as malignant using semiquantitative data from ^18^F-FDG PET/CT. The final histopathological diagnosis was adrenal malignant transformation of angiomyolipoma

The analysis included 102 unilateral or bilateral nonsecreting adrenal tumours in 85 patients. Of the 85 patients, 17 had bilateral adrenal lesions. Based on final pathology, of 70 benign adrenal tumours, 54 were adenomas (Fig. 1), 6 tuberculosis, 4 adrenal hyperplasia, 4 myelolipomas, and 2 phaeochromocytomas. Of 30 metastatic malignant tumours, 17 were from colon carcinoma, 10 from lung cancer (Fig. 2), 1 from melanoma, 1 from gastric cancer, and 1 adrenal lymphoma. There were two primary adrenal tumours: one adrenal cortical carcinoma and a very rare entity: angiomyolipoma with malignant potential (Fig. 3).

### PET/CT in differentiating malignant from benign adrenal lesions

**Fig. 4:**
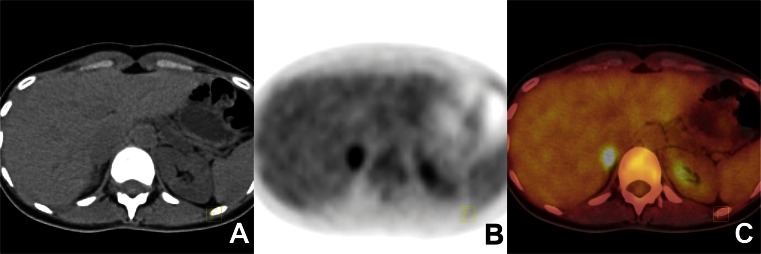
Phaeochromocytoma of the right adrenal gland. False-positive ^18^F-FDG PET/CT findings in a 38-year-old woman with lung cancer (**a** CT image, **b** PET image, **c** fused PET/CT image). The ^18^F-FDG PET/CT image shows increased uptake of ^18^F-FDG in a right adrenal tumour. The SUVmax of the tumour was 8.4 and the attenuation value 41 HU; the liver SUVmax was 3.0. The final histopathological diagnosis was benign phaeochromocytoma

**Fig. 5:**
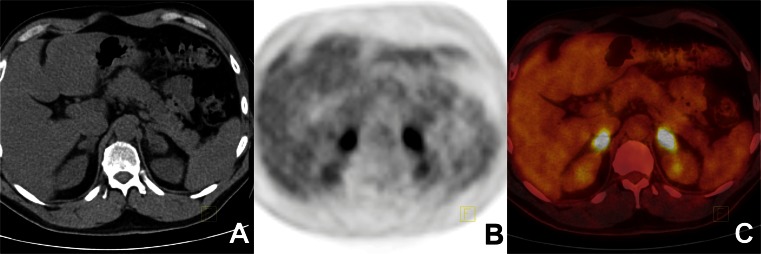
Bilateral adrenal tuberculosis. False-positive ^18^F-FDG PET/CT findings in a 62-year-old man with a history of lung cancer (**a** CT image, **b** PETimage, **c** fused PET/CTimage). The ^18^F-FDGPET/CTimage shows enlarged adrenal tumours with bilateral intense uptake of ^18^F-FDG. The SUVmax of the left adrenal tumour was 12.1 and the attenuation value 33 HU, and the SUVmax value of the right adrenal tumour was 14.4 and the attenuation value 29 HU; the liver SUVmax was 2.4. The final histopathological diagnosis was bilateral adrenal tuberculosis

Malignant adrenal tumours had a significantly higher mean SUVmax (13.0 ± 7.1 vs. 3.7 ± 3.0; *p* < 0.001), higher T/L SUVmax ratio (4.2 ± 2.6 vs. 1.0 ± 0.9, *p* < 0.001), higher CT attenuation value (31.91 ± 16. 7 HU vs. 0.2 ± 25.8 HU, *p* < 0.001) and a greater diameter (43.6 mm ± 23.7 vs. 25.6 mm ± 13.3, *p* < 0.001) than benign lesions. Six false-positive results were observed in our series: four adrenal tuberculosis and two benign phaeochromocytomas (Figs. 4 and 5). The benign lesions related to tuberculosis showed a hypermetabolic pattern with T/L SUVmax ratios in the range 3.2 – 5.2. The ^18^F-FDG PET/CT scan showed intense uptake of ^18^F-FDG in bilateral tumours in two patients (SUVmax 12.3 – 19) with T/L SUVmax ratios in the range 3.2 – 5.2 and attenuation values in the range 32 – 37 HU, findings that suggested malignancy. Interestingly, in the third patient with bilateral adrenal tuberculosis, ^18^F-FDG PET/CT showed lower ^18^F-FDG uptake with SUVmax 3.2 and 4.8) and T/L SUVmax ratios 0.9 and 1.3.

**Fig. 6:**
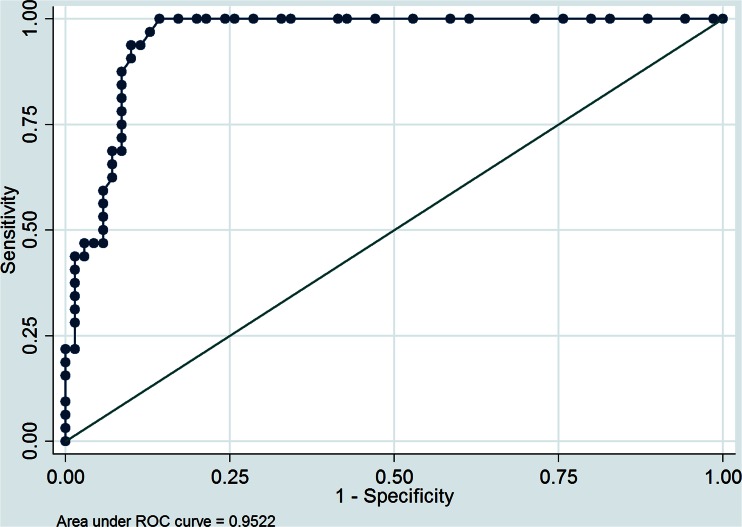
ROC curve for the ability of adrenal SUVmax to predict malignancy

**Fig. 7:**
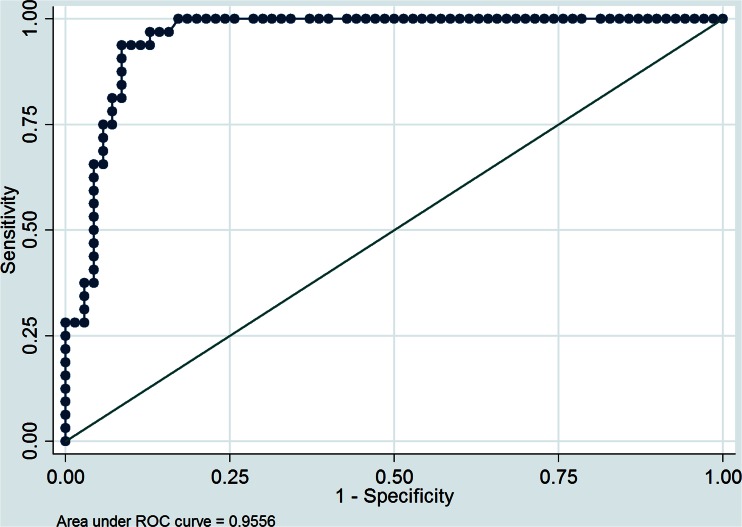
ROC curve for the ability of the T/L SUVmax ratio to predict malignancy

Significant predictors of malignancy in univariate logistic regression analysis were: T/L SUVmax ratio (OR 3.7, 95 % CI 2.26 – 5.99; *p* < 0.001), adrenal SUVmax (OR 1.5, 95 % CI 1.25 – 1.71; *p* < 0.001), attenuation value (OR 1.11, 95 % CI 1.06 – 1.16; *p* < 0.001) and diameter (OR 1.06, 95 % CI 1.03 – 1.09; *p* < 0.001). Based on the ROC analysis, an adrenal SUVmax >5.2, T/L SUVmax ratio >1.53, attenuation value >24 HU and tumour diameter >30 mm were chosen as optimal cut-off values for differentiating malignant from benign tumours (Figs. 6 and 7). The sensitivities and specificities of these cut-off values were: 91 % (95 % CI 85 – 96 %) and 90 % (95 % CI 84 – 96 %), 94 % (95 % CI 89 – 99 %) and 91 % (95 % CI 86 – 97 %), 88 % (95 % CI 81 – 94 %) and 81 % (95 % CI 74 – 89 %), and 49 % (95 % CI 37 – 57 %) and 89 % (95 % CI 82 – 98 %), respectively. The positive predictive values (PPV) and negative predictive values (NPV) of these cut-off values were: 81 % (95 % CI 73 – 88 %) and 96 % (95 % CI 91 – 99.5 %), 83 % (95 % CI 76 – 91 %) and 97 % (95 % CI 94 – 100 %), 65 % (95 % CI 56 – 75 %) and 79 % (95 % CI 71 – 87 %), and 68 % (95 % CI 59 – 77 %) and 93 % (95 % CI 89 – 98 %), respectively.

A threshold value of 10 HU, generally accepted as the cut-off value for differentiating adrenal tumours in our study population, had a sensitivity of 94 % (95 % CI 89 – 99 %) and specificity of 67 % (95 % CI 58 – 76 %). The PPV and NPV for diagnosing adrenal adenoma were 57 % (95 % CI 47 – 66 %) and 96 % (95 % CI 92 – 99.8 %), respectively. The AUC for adrenal SUVmax >5.2 was 0.96 (95 % CI 0.91 – 0.99 %), for T/L SUVmax ratio >1.53 was 0.96 (95 % CI 0.92 – 0.99 %, for attenuation value >24 HU was 0.81 (95 % CI 0.81 – 0.95 %), and for tumour diameter >30 mm was 0.77 (95 % CI 0.66 – 0.87 %). Based on the AUC, adrenal SUVmax and T/L SUVmax ratio exhibited the highest and comparable diagnostic performance for predicting malignant lesions. Tumour diameter had the lowest AUC and was a poor predictor of malignant disease.

Multivariate logistic regression model revealed that T/L SUVmax ratio was an independent predictive factor for malignancy (OR 126.0, 95 % CI 10.5 – 1512.9, *p* < 0.001). CT attenuation value >24 HU and tumour diameter >30 mm were not independent predictive factors (*p* > 0.1)

## Discussion

^18^F-FDG PET/CT is a very useful tool for differentiating benign from malignant adrenal lesions in patients with proven malignancy with an overall sensitivity between 74 % and 100 %, and specificity between 69 % and 100 % [17]. However, many studies addressing this issue have been limited by their retrospective design, small sample size, heterogeneous patient population, or analysis based on PET imaging alone. In published studies the final diagnosis was based mostly on clinical or radiological follow-up after 6 – 24 months [3, 5, 7, 10–13, 16]. Only some studies used a reference standard based on final pathology obtained in all patients [5]. The earlier studies showed that the use of qualitative (visual) data for characterizing adrenal tumours may be more helpful than SUVmax or SUVmean. The authors of the largest study of the quantitative and qualitative value of ^18^F-FDG PET/CT suggested that qualitative analysis alone should suffice for differentiation between benign and malignant adrenal tumours and the routine use of SUVmean or SUVmax may be unnecessary [11]. However, the quantitative criteria seem to be subjective and to yield inconsistent results especially for borderline cases. On the other hand critics of quantitative methods contend that SUV measurements are variable due to the type of ROI, partial volume effects and many other factors including the patient’s body composition, plasma glucose level, image noise and image reconstruction method used [3, 18].

Over the last several years, PET/CT techniques have evolved resulting in more sensitive cameras. The combination of higher resolution PET cameras, and new reconstruction and correction algorithms used in the acquisition have provided the required maximum image quality for precise SUV measurements. Due to advances in technology quantitative analysis with PET/CT has been recognized as providing an objective, more accurate, and less observer-dependent measure for characterizing adrenal tumours than visual assessment alone [6, 18, 19]. In published studies authors have suggested adrenal SUVmax cut-off values of 2.3, 2.5, 2.7, 3.1, 3.4 and 3.9, but it has been well recognized that absolute adrenal SUVmax or SUVmean threshold values that could reliably differentiate all benign from malignant adrenal tumours do not exist [10–12, 15, 16, 20, 21]. The SUVmax cut-off >3.9 yielded a sensitivity of 96 % and a specificity of 82 % [15].

Recently the use of the T/L SUVmax ratio has been recommended to improve the reproducibility and performance of ^18^F-FDG PET/CT. The use of liver activity as internal control that may enable monitoring of the reliability of SUV measurements and in this way the effects of many external factors, can be omitted [5, 15, 16]. Different T/L SUVmax ratio cut-off values (1, 1.37, 1.45, 1.8) have been proposed for differentiating benign from malignant tumours with good sensitivity but weaker specificity [5, 7, 10, 11, 15, 16, 20]. In one study a T/L SUV ratio >1.37 yielded a sensitivity of 96 % and specificity of 100 % and had a greater ability to differentiate benign adenomas from adrenal metastases than SUVmax [15]. In another study, the best correlation between ^18^F-FDG PET/CT imaging and histological evaluation of adrenal tumours was found using a T/L SUVmax ratio cut-off >1.0 [16]. This threshold was more accurate in differentiating the tumour type than SUVmax or visual interpretation alone, yielding a sensitivity of 97 % and a specificity of 92 % [16]. Similarly, in our study we found that ^18^F-FDG PET/CT was highly efficient in distinguishing malignant from benign adrenal tumours. In our series a T/L SUVmax ratio cut-off of 1.53 corresponded to a sensitivity of 93.8 % and a specificity of 91.4 %, whereas an adrenal SUVmax cut-off of 5.2 corresponded to a sensitivity of 90.6 % and a specificity of 90 %. In evaluating adrenal tumours a T/L SUVmax ratio of 1.53 yielded high sensitivity, specificity, NPV comparable to those using an adrenal SUVmax of 5.2. Moreover, although the use of liver activity as an internal control should correct for some of the variables that affect SUV measurements, no significant differences in terms of diagnostic accuracy were observed between the two parameters, but T/L SUV_max_ ratio was found to be an independent predictor of malignancy. In the ROC curve analysis tumour diameter had the lowest AUC. The diagnostic threshold for a diameter >30 mm yielded a sensitivity of 48.9 % and a specificity of 88.6 % so was a poor predictor of malignancy.

The major cause of false-positive results may vary among studies. In previous studies false-positive ^18^F-FDG PET/CT findings were mainly adrenal adenomas and benign phaeochromocytomas. It has been suggested that the functional state of an adenoma could be a factor determining the intensity of ^18^F-FDG metabolism, but others have found a lack of correlation between hormonal hypersecretion and ^18^F-FDG uptake. Several studies have shown that tuberculosis can produce high ^18^F-FDG uptake and limit the specificity of ^18^F-FDG PET/CT scans by mimicking metastases [11, 12, 16, 22, 23].

The finding of our study confirm those of previous studies that ^18^F-FDG accumulates in some benign adrenal masses. In our series a T/L SUVmax ratio threshold of >1.53 yielded six false-positive results: four adrenal tuberculosis and two benign phaeochromocytomas. The benign lesions related to tuberculosis showed a hypermetabolic pattern. In one patient with bilateral adrenal tuberculosis, ^18^F-FDG- PET/CT showed low uptake of ^18^F-FDG, which could have been the result of a lack of granulomatous inflammation due to the local suppressive effect of steroids secreted in the adrenal cortex. Four false-positive results underscore the necessity for keeping adrenal tuberculosis in mind as a possible differential diagnosis in bilateral adrenal masses in regions where tuberculosis is endemic. In previous studies, false-negative results have been recognized because of a small lesion size (<8 mm), the low ^18^F-FDG avidity of certain cancer types (e.g. renal cell cancer, neuroendocrine tumours, bronchoalveolar carcinoma), and metastases with haemorrhage and/or necrosis [12, 14, 16, 24]. Some authors have speculated that false-negative adrenal metastases might be the result of suppressive effects of prior chemotherapy on tumour glucose metabolism [14, 16]. In this study the T/L SUVmax ratio threshold of >1.53 yielded two false-negative results for adrenal metastasis from colon carcinoma and adrenal carcinoma with SUVmax 4.2 and 4.6 and T/L SUVmax ratios 1 and 1.12, respectively. One of these patients had a history of chemotherapy received 4 months before examination, and the other had a large tumour with necrosis.

Some studies have indicated that the accuracy of ^18^F-FDG PET/CT might be improved by combining the PET parameters with the attenuation value from the unenhanced CT scan [10, 12, 25]. In one study the use of a mean CT attenuation of >10 HU combined with either of the PET criteria SUVmax >3.1 or T/L SUV ratio >1.0 improved the performance of PET/CT in characterizing adrenal tumours. A combined T/L SUVmax ratio of >3.1 and a mean attenuation of >10 HU had a sensitivity of 97.3 % and a specificity of 86.2 %, a combined T/L SUVmax ratio of >1.0 and a mean attenuation of >10 HU had a sensitivity of 97.3 % and a specificity of 74.1 %. The accuracies of these threshold combinations (90.5 % and 83.2 %, respectively) were significantly different. [10]. In another recent study showed better sensitivity and accuracy with a combined T/L SUVmax ratio of >1.3 and HU >18. The sensitivity, specificity and accuracy of this method for predicting malignancy were 97.7 %, 81.2 % and 93.4 %, respectively [25].

It has been reported that lipid-rich adenoma may be differentiated from lipid-poor adenoma and metastasis using a cut-off value of 10 HU on unenhanced CT images. However, limitations of this method have been reported in oncology patients. Some authors found that unenhanced CT has a low specificity and sensitivity, which leads to false-positive and false-negative results. Based on a threshold of >10 HU, CT misdiagnosed 21 % of true adenomas as metastases and misdiagnosed 11 % of metastases as adenomas [26]. In our study, a CT threshold of 10 HU for differentiating adrenal tumours had a sensitivity of 93.8 %, a specificity of 67.1 %, a PPV of 56.6 % and a NPV of 95.9 %.

In the multivariate analysis including adrenal SUVmax, T/L SUVmax ratio, CT attenuation value and tumour diameter categorized according to the established cut-off values as predictors only T/L SUVmax ratio was a predictor of malignancy. In contrast to previously published data, combined T/L SUVmax ratio and HU attenuation value from unenhanced CT images did not improve the characterization of adrenal tumours in our series. Only the T/L SUVmax ratio was found to be an independent predictor of malignancy.

It is well recognized that selection of the reference standard is critical for the validity of a study of a test’s accuracy and the definition of the diagnostic threshold forms part of the reference standard. The majority of published studies have mainly been based on imperfect reference standards such as assessing the change in tumour size over a follow-up of 6 – 24 months [3, 10–14]. Errors resulting from the use of an imperfect reference standard translate into poorer accuracy in the evaluation of a test’s diagnostic performance.

To the best of our knowledge, this is the second largest study with histology confirmation comparing the ability of ^18^F-FDG PET/CT to characterize adrenal lesions. However the study had some limitations. First, the study was retrospective and there was unavoidable selection bias. The second limitation was the small and heterogeneous sample size: patients had a variety of different malignancies and therapy regimens.

## Conclusion

Based on our results the use of quantitative parameters of ^18^F-FDG PET/CT led to high sensitivity, specificity and NPV in diagnosing adrenal tumours in oncological patients. Diagnostic accuracies of adrenal SUVmax and T/L SUVmax ratio were comparable, but T/L SUVmax ratio was an independent predictor of malignancy. Further large multicentre prospective studies based on histology as the reference standard are needed to confirm our results and determine whether they can be applied to healthy patients with adrenal incidentalomas.

## References

[1] Lenert JT, Barnett CC, Kudelka AP, Sellin RV, Gagel RF, Prieto VG (2001). Evaluation and surgical resection of adrenal masses in patients with a history of extra-adrenal malignancy. Surgery.

[2] Abrams HL, Spiro R, Goldstein N (1950). Metastases in carcinoma: analysis of 1,000 autopsied cases. Cancer.

[3] Boland GW, Dwamena BA, Jagtiani Sangwaiya M, Goehler AG, Blake MA, Hahn PF (2011). Characterization of adrenal masses by using FDG PET: a systematic review and meta-analysis of diagnostic test performance. Radiology.

[4] Blake MA, Prakash P, Cronin CG (2010). PET/CT for adrenal assessment. AJR Am J Roentgenol.

[5] Groussin L, Bonardel G, Silvéra S, Tissier F, Coste J, Abiven G (2009). 18F-fluorodeoxyglucose positron emission tomography for the diagnosis of adrenocortical tumors: a prospective study in 77 operated patients. J Clin Endocrinol Metab.

[6] Elaini AB, Shetty SK, Chapman VM, Sahani DV, Boland GW, Sweeney AT (2007). Improved detection and characterization of adrenal disease with PET-CT. Radiographics.

[7] Tessonnier L, Sebag F, Palazzo FF, Colavolpe C, De Micco C, Mancini J (2008). Does 18F-FDG PET/CT add diagnostic accuracy in incidentally identified non-secreting adrenal tumours?. Eur J Nucl Med Mol Imaging.

[8] Chong S, Lee KS, Kim HY, Kim YK, Kim BT, Chung MJ (2006). Integrated PET-CT for the characterization of adrenal gland lesions in cancer patients: diagnostic efficacy and interpretation pitfalls. Radiographics.

[9] Perri M, Erba P, Volterrani D, Guidoccio F, Lazzeri E, Caramella D (2011). Adrenal masses in patients with cancer: PET/CT characterization with combined CT histogram and standardized uptake value PET analysis. AJR Am J Roentgenol.

[10] Brady MJ, Thomas J, Wong TZ, Franklin KM, Ho LM, Paulson EK (2009). Adrenal nodules at FDG PET/CT in patients known to have or suspected of having lung cancer: a proposal for an efficient diagnostic algorithm. Radiology.

[11] Boland GW, Blake MA, Holalkere NS, Franklin KM, Ho LM, Paulson EK (2009). PET/CT for the characterization of adrenal masses in patients with cancer: qualitative versus quantitative accuracy in 150 consecutive patients. AJR Am J Roentgenol.

[12] Metser U, Miller E, Lerman H, Lievshitz G, Avital S, Even-Sapir E (2006). 18F-FDG PET/CT in the evaluation of adrenal masses. J Nucl Med.

[13] Park BK, Kim CK, Kim B, Choi JY (2007). Comparison of delayed enhanced CT and 18FFDG PET/CT in the evaluation of adrenal masses in oncology patients. J Comput Assist Tomogr.

[14] Vikram R, Yeung HD, Macapinlac HA, Iyer RB (2008). Utility of PET/CT in differentiating benign from malignant adrenal nodules in patients with cancer. AJR Am J Roentgenol.

[15] Watanabe H, Kanematsu M, Goshima S, Kondo H, Kawada H, Noda Y (2013). Adrenal-to-liver SUV ratio is the best parameter for differentiation of adrenal metastases from adenomas using (18)F-FDG PET/CT. Ann Nucl Med.

[16] Gratz S, Kemke B, Kaiser W, Heinis J, Behr TM, Höffken H (2010). Incidental non-secreting adrenal masses in cancer patients: intra-individual comparison of 18F-fluorodeoxyglucose positron emission tomography/computed tomography with computed tomography and shift magnetic resonance imaging. J Int Med Res.

[17] Wong KK, Arabi M, Bou-Assaly W, Marzola MC, Rubello D, Gross MD (2012). Evaluation of incidentally discovered adrenal masses with PET and PET/CT. Eur J Radiol.

[18] Boellaard R (2009). Standards for PET image acquisition and quantitative data analysis. J Nucl Med.

[19] Bockisch A, Freudenberg LS, Schmidt D, Kuwert T (2009). Hybrid imaging by SPECT/CT and PET/CT proven outcomes in cancer imaging. Semin Nucl Med.

[20] Okada M, Shimono T, Komeya Y, Ando R, Kagawa Y, Katsube T (2009). Adrenal masses: the value of additional fluorodeoxyglucose-positron emission tomography/computed tomography (FDG-PET/CT) in differentiating between benign and malignant lesions. Ann Nucl Med.

[21] Ansquer C, Scigliano S, Mirallié E, Taïeb D, Brunaud L, Sebag F (2010). 18F-FDG PET/CT in the characterization and surgical decision concerning adrenal masses: a prospective multicentre evaluation. Eur J Nucl Med Mol Imaging.

[22] Rao SK, Caride VJ, Ponn R, Giakovis E, Lee SH (2004). F-18 fluorodeoxyglucose positron emission tomography-positive benign adrenal cortical adenoma: imaging features and pathologic correlation. Clin Nucl Med.

[23] Li YJ, Cai L, Sun HR, Gao S, Bai RJ (2008). Increased FDG uptake in bilateral adrenal tuberculosis appearing like malignancy. Clin Nucl Med.

[24] Kumar R, Xiu Y, Yu JQ, Takalkar A, El-Haddad G, Potenta S (2004). 18F-FDG PET in evaluation of adrenal lesions in patients with lung cancer. J Nucl Med.

[25] Cho AR, Lim I, Im Il N, Choe D, Park J, Kim B (2011). Evaluation of adrenal masses in lung cancer patients using F-18FDG-PET/CT. Nucl Med Mol Imaging.

[26] Porte HL, Ernst OJ, Delebecq T, Métois D, Lemaitre LG, Wurtz AJ (1999). Is computed tomography guided biopsy still necessary for the diagnosis of adrenal masses in patients with resectable non-small-cell lung cancer?. Eur J Cardiothorac Surg.

